# Volumetric Bone Mineral Density Assessed by Dual-Energy CT Predicts Bone Strength Suitability for Cementless Total Knee Arthroplasty

**DOI:** 10.3390/medicina61071305

**Published:** 2025-07-20

**Authors:** Dong Hwan Lee, Dai-Soon Kwak, Sheen-Woo Lee, Yong Deok Kim, Nicole Cho, In Jun Koh

**Affiliations:** 1 Department of Orthopaedic Surgery, Yeouido St. Mary’s Hospital, Seoul 07345, Republic of Korea; ldh850606@naver.com; 2 Department of Orthopaedic Surgery, College of Medicine, The Catholic University of Korea, Seoul 06591, Republic of Korea; seraph622@naver.com; 3 Catholic Institute for Applied Anatomy, Department of Anatomy, College of Medicine, The Catholic University of Korea, Seoul 06591, Republic of Korea; daisoon@catholic.ac.kr; 4 Department of Radiology, Eunpyeong St. Mary’s Hospital, Seoul 03312, Republic of Korea; leesw1@catholic.ac.kr; 5 Joint Replacement Center, Eunpyeong St. Mary’s Hospital, Seoul 03312, Republic of Korea; 6 Hackensack Meridian School of Medicine, 123 Metro Blvd, Nutley, NJ 07100, USA; nicole.cho@hmhn.org

**Keywords:** bone quality, dual-energy CT (DECT), volumetric bone mineral density (vBMD), cementless, total knee arthroplasty

## Abstract

*Background and Objectives*: Adequate bone quality is essential for promoting initial bone ingrowth and preventing early migration during cementless total knee arthroplasty (TKA). However, gold-standard criteria for identifying suitable bone strength have yet to be established. Dual-energy computed tomography (DECT)-based volumetric bone mineral density (vBMD) is an emerging tool for assessing bone quality. This study aimed to determine whether DECT-derived vBMD can accurately predict suitable bone strength for cementless TKA. *Materials and Methods*: A total of 190 patients undergoing primary TKA with a standardized posterior-stabilized implant were prospectively enrolled. Prior to TKA, DECT-derived vBMD was measured in the femoral box region. Actual bone strength was evaluated using an indentation test on resected femoral box specimens. Correlation and linear regression analyses were performed to assess the relationship between DECT vBMD and actual bone strength. Receiver operating characteristic (ROC) curve analysis and area under the curve (AUC) calculations were used to determine the optimal cut-off value and diagnostic accuracy of DECT vBMD in identifying candidates suitable for cementless TKA. *Results*: DECT-derived vBMD exhibited a strong correlation with actual bone strength (correlation coefficient = 0.719, *p* < 0.01), while linear regression analysis revealed a moderate association (R^2^ = 0.51, *p* < 0.01). In addition, it demonstrated excellent diagnostic performance in predicting adequate bone quality for cementless TKA, yielding an AUC of 0.984, with a sensitivity of 91.9% and a specificity of 92.0%. *Conclusions*: DECT-derived vBMD is a reliable and accurate tool for assessing bone strength around the knee and predicting the suitable bone quality for cementless TKA.

## 1. Introduction

Recent innovations in technology, coupled with an escalation in the population of obese individuals, have contributed to a demographic shift, leading to a rise in TKA procedures among young, active individuals [1,2,3]. Consequently, the prevalence of aseptic loosening has increased, rendering it a predominant cause of revision TKA [4,5]. In response, enhancements in the biological fixation of cementless TKA implants have yielded favorable early to mid-term clinical results and long-term survivorship [6,7,8,9,10]. The success of cementless TKA largely depends on achieving initial rigid fixation, which is profoundly affected by bone quality [11,12]. However, clear selection criteria for determining the ideal candidates for cementless TKA have not been defined.

Central dual-energy X-ray absorptiometry (DXA) bone mineral density (BMD), including measurements of the pelvic bone and lumbar spine, has been the most widely used method for evaluating bone quality. However, recent studies have reported that central BMD is not a reliable surrogate for assessing bone quality in peripheral joints, highlighting the need for joint-specific assessments, particularly in the knee joint [13,14,15]. Recently, volumetric BMD (vBMD) assessed by dual-energy computed tomography (DECT) has been introduced. DECT utilizes the differences in X-ray absorption among various materials, and this energy-based approach enables superior tissue differentiation compared to conventional CT [16]. In addition, DECT provides three-dimensional volumetric data, allowing for spatial assessment of BMD, which is particularly effective for evaluating local bone quality [17]. DECT vBMD showed a high correlation with BMD measurements obtained via DXA [18,19] and has been reported to offer superior consistency, reliability, and diagnostic accuracy [19,20,21]. However, the correlation between DECT-derived vBMD and the actual bone strength around the knee joint remains unclear, and its efficacy in screening candidates for cementless TKA has yet to be validated.

This study was conducted to determine (1) whether DECT-derived vBMD correlates with the actual bone strength of the knee, and (2) whether DECT-derived vBMD can predict the suitability of bone strength for cementless TKA. We hypothesized that DECT vBMD would show a correlation with the actual bone strength of the knee. It was also hypothesized that DECT vBMD would accurately identify the ideal candidates for cementless TKA.

## 2. Materials and Methods

### 2.1. Study Participants

This prospective study included 190 knees undergoing posterior-stabilized (PS) TKA using the Triathlon^®^ system (Stryker Inc., Mahwah, NJ, USA) between May 2022 and May 2024. Institutional review board approval was obtained (PC22OISI0068), and informed consent was collected from all participants. Only patients with primary osteoarthritis who underwent preoperative DECT and agreed to participate were included. Among the 190 patients, 155 (82%) were female. The mean age was 68.1 ± 5.4 years (range, 53–86), with a mean height of 155.5 ± 7.0 cm, weight of 65.7 ± 9.6 kg, and body mass index of 27.2 ± 3.4 kg/m^2^. Mean preoperative DECT vBMD values were 55.9 ± 31.9 mg/cm^3^ (axial image), 50.2 ± 26.9 mg/cm^3^ (sagittal image), and 54.6 mg/cm^3^ (coronal image) (Table 1).

### 2.2. Dual-Energy CT Imaging Protocol and Analysis

**Figure 1:**
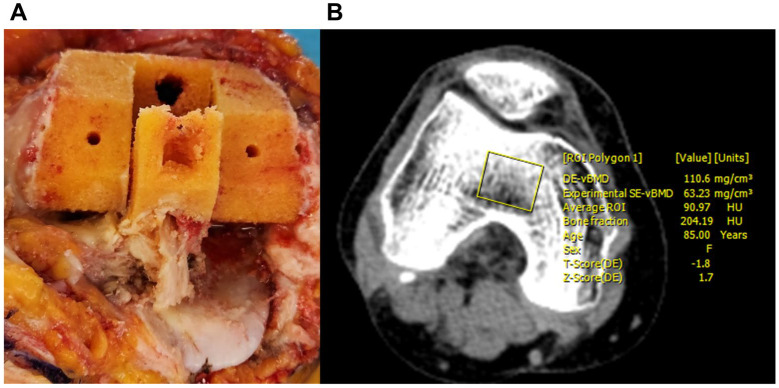
Collection of a box bone specimen during femoral box preparation for posterior-stabilized TKA (**A**). Measurement of DECT-fderived vBMD with the region of interest (ROI) defined to match the resected bone area (**B**).

All CT scans were performed one week prior to surgery with patients positioned supine and head first, using a third-generation dual-source CT system (SOMATOM Force; Siemens Healthineers, Forchheim, Germany) in dual-energy mode. Tube A was set at 80 kVp and tube B at Sn150 kVp (0.64 mm tin filter, 180 mAs). The scan range extended from the femoral head to the midfoot without contrast enhancement. Three datasets (90 kVp, Sn150 kVp, and a 0.5:0.5 blended average) were obtained in a 0.8 mm slice thickness, 512 × 512 matrix images using a high-resolution soft tissue kernel (Qr40), and they were transferred to a dedicated workstation (eXamine, software version VB80D) for quantitative analysis. Two orthopedic surgeons with extensive experience and fellowship training in knee surgery manually defined regions of interest (ROIs) that matched the resected femoral box area while avoiding cortical bone, vessels, and artifacts. The software automatically calculated vBMD (mg/cm^3^) using a phantomless internal calibration algorithm. Scans with significant motion or artifacts were excluded from analysis (Figure 1). We used a two-way random-effects model with absolute agreement to find the intraclass correlation coefficient (ICC) in order to check the inter-observer reliability. The obtained ICC value of 0.91 indicated excellent reliability for our vBMD measurements. The radiation dose of DECT in this study showed a mean CTDIvol (CT dose index volume) of 5.08 mGy.

### 2.3. Bone Strength Assessment

**Figure 2:**
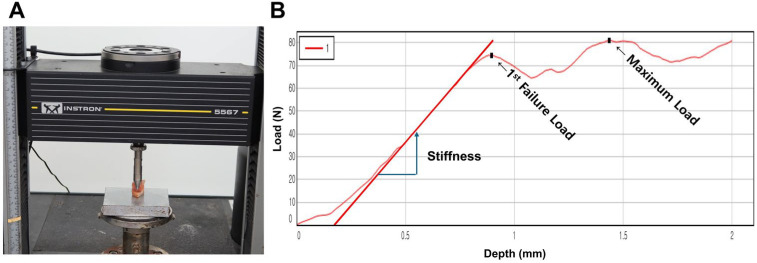
Preparation of the bone specimen for indentation testing (**A**). The Instron testing system calculated stiffness, first failure load, and maximum failure load from the load–displacement curve (**B**).

To evaluate bone strength, an indentation test was performed as previously validated in the literature [13,22,23,24]. Bone specimens were obtained during the box preparation in TKA (Figure 1) and stored at −70 °C until testing. Prior to the experiment, specimens were thawed and trimmed to a uniform thickness of 6 mm using a linear precision saw (IsoMet 5000; Buehler, Lake Bluff, IL, USA) with dual diamond blades. Two diamond blades were securely fixed at a 6 mm interval and rotated at high speed to cut the specimens, resulting in parallel cutting surfaces. During the cutting process, saline solution was continuously irrigated to prevent thermal damage. Trabecular bone alignment was standardized by securing bone fragments based on the box resection geometry obtained during TKA surgery and performing parallel cuts accordingly. Although it is challenging to account for all microscopic trabecular alignments since all specimens were uniformly positioned based on the box resection configuration, consistent positioning and parallel cutting were maintained at identical locations for all specimens. Each specimen was mounted on a servohydraulic testing machine (Instron 5567; Norwood, MA, USA), and indentation testing was performed using a flat punch indenter with a diameter of 6 mm (contact area: 28.3 mm^2^). The indenter was positioned under a preload of 2 N to establish contact with the specimen surface. Indentation was then performed to a depth of 2 mm at a rate of 1.0 mm/min. The test continued until both the first failure load and the maximum load were obtained. Load and displacement data were collected at a sampling rate of 30 Hz and analyzed using the manufacturer’s software (Instron Bluehill v4.23) (Figure 2). The first failure load, defined as the initial deviation from linearity in the load–displacement curve, was 59.5 ± 38.2 N (range, 6.7–202.5 N) (Table 2).

### 2.4. Definition of Suitability for Cementless TKA

**Figure 3:**
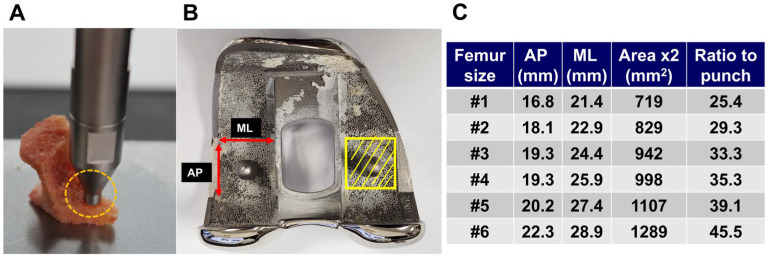
The flat punch used in the indentation test had a contact area of 28.3 mm^2^ (**A**). The distal femoral component area was calculated as 2 × (anterior-posterior [AP] × mediolateral [ML]) dimensions (**B**). The Estimated Withstanding Strength (EWS) was defined as the product of the first failure load and the ratio of the distal femoral component area to the punch area (**C**).

To define the Minimum Required Strength (MRS) for cementless TKA, we adopted a threshold equivalent to 2.5 times the patient’s body weight. This value is supported by previous in vivo biomechanical studies that demonstrated that peak joint contact forces in the knee during routine activities such as walking can reach up to 2.5 to 3 times body weight following TKA [25,26]. In addition, the Estimated Withstanding Strength (EWS) was defined as the adjusted load, calculated by multiplying the first failure load obtained from the indentation test by the ratio of the femoral component’s distal surface area to the punch cross-sectional area (28.3 mm^2^) (Figure 3). Patients with EWS exceeding the MRS were categorized as ‘cementless suitable,’ and those below the threshold as ‘cemented mandatory.’ The corresponding DECT vBMD values were analyzed to determine the diagnostic threshold for cementless TKA candidacy.

### 2.5. Statistical Analysis

All statistical analyses were performed using SPSS version 21 (IBM Corp., Armonk, NY, USA). A *p*-value < 0.05 was considered statistically significant. Linear regression analysis was used to examine the correlation between DECT vBMD and the first failure load. Receiver operating characteristic (ROC) curve analysis was conducted to determine the diagnostic cut-off value of DECT vBMD for predicting suitability for cementless TKA. Sensitivity, specificity, and area under the curve (AUC) were calculated. A post hoc power analysis was conducted based on the observed effect size (correlation coefficient = 0.719). With a total sample size of 190 knees and a significance level of 0.05, the calculated statistical power exceeded 0.99, indicating that the study was adequately powered to detect the observed relationship between DECT vBMD and bone strength.

## 3. Results

**Figure 4:**
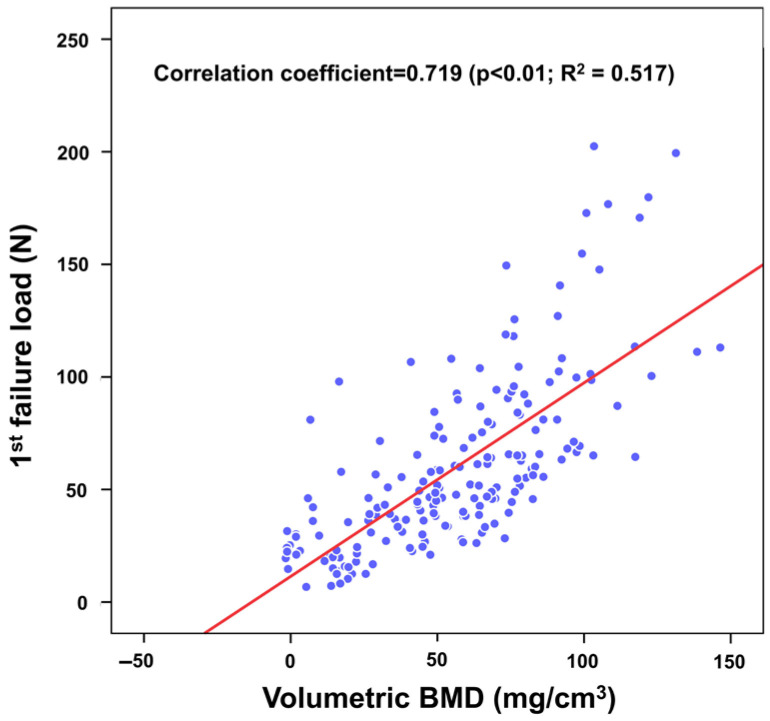
Correlation analysis between DECT-derived vBMD and the first failure load of the box bone. The correlation coefficient was 0.719 (*p* < 0.001), and the linear regression model demonstrated an R^2^ value of 0.51.

DECT-derived vBMD showed a strong correlation with the first failure load of the box bone as measured by the indentation test. The correlation coefficient was 0.719 (*p* < 0.01), and linear regression analysis revealed a moderate correlation, with an R^2^ value of 0.51 (*p* < 0.01) (Figure 4).

**Figure 5:**
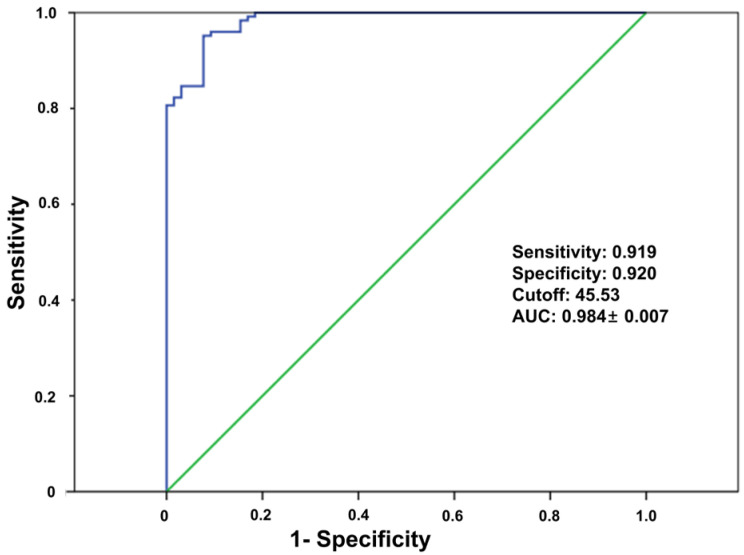
ROC curve analysis of DECT-derived vBMD for predicting adequate bone strength for cementless TKA. The optimal cut-off value was 45.53 g/cm^3^, with an AUC of 0.984 ± 0.007, sensitivity of 91.9%, and specificity of 92.0%.

DECT-derived vBMD accurately predicted the suitability of bone strength for cementless TKA. The ROC curve analysis yielded an AUC of 0.984, with a sensitivity of 91.9% and a specificity of 92%. The optimal DECT vBMD cut-off value for identifying suitable candidates for cementless TKA was 45.53 g/cm^3^ (Figure 5).

## 4. Discussion

With the increasing prevalence of TKA among younger, more active patients and ongoing advancements in cementless implant technology, the use of cementless TKA has significantly increased [7,27]. However, a gold standard for suitable bone strength, which is crucial to prevent early failure, has not yet been established [28,29]. In this study, we aimed to investigate whether the recently introduced volumetric bone mineral density (vBMD) measured by dual-energy computed tomography (DECT) correlates with actual bone strength around the knee, and whether it can accurately predict adequate bone strength for cementless TKA.

Our findings suggest that DECT-derived vBMD accurately predicts the actual bone strength around the knee joint. In this study, correlation and linear regression analyses demonstrated a strong association between DECT vBMD and the first failure load (correlation coefficient = 0.719, *p* < 0.001, R^2^ = 0.51). DECT captures data at two distinct energy levels, enabling superior tissue differentiation by exploiting the differential X-ray absorption properties of various materials [16]. This technology provides three-dimensional vBMD measurements without the need for calibration phantoms, allowing precise spatial assessment of local bone quality [17]. Previous studies have reported a high correlation between DECT-derived vBMD and BMD values measured by DXA and QCT, supporting this technique’s utility in osteoporosis assessment [21,30]. However, to date, no study has directly examined the correlation between DECT-derived vBMD and actual bone strength. On the other hand, DECT can be combined with the iterative metal artifact reduction (iMAR) algorithm, facilitating the simultaneous assessment of implant loosening and bone quality around the implant. This makes it particularly valuable for patients undergoing evaluation for revision TKA [31]. Taken together with previous reports, our findings indicate that DECT is a pragmatic and accurate tool for assessing bone quality, both in the context of primary and revision TKA.

The results of this study demonstrated that DECT-derived vBMD is an excellent screening tool for predicting adequate bone strength for cementless TKA. ROC curve analysis yielded an outstanding AUC of 0.984, with a sensitivity of 91.9% and a specificity of 92.0%. In addition, the optimal DECT vBMD cut-off value for identifying the suitable bone strength for cementless TKA was determined to be 45.53 g/cm^3^. Our findings are consistent with previous studies that have reported the excellent diagnostic value of DECT vBMD for diagnosing osteoporosis in the spine [21,32] and knee joint [20]. Meanwhile, recent studies, however, have indicated that central BMD measured by DXA may not serve as a reliable surrogate for assessing bone quality in the knee joint [13]. Our findings, in conjunction with previous studies, highlight the need for a knee-joint-specific tool to assess local bone quality, and support the use of DECT-derived vBMD as a suitable alternative to address this need. This study represents a preliminary investigation into the threshold of bone strength required for successful cementless TKA, and further research is warranted to establish more precise and clinically applicable criteria.

This study has several limitations. First, the study population was limited, comprising 82% female patients and exclusively Asian participants. This reflects the demographic characteristics of TKA recipients in our country [33]. As bone specimens were required, inclusion was limited to TKA patients, making this limitation unavoidable. It is well established that bone mineral density and bone strength vary according to ethnicity and gender, and substantial ethnic differences have been reported even in knee alignment [34,35,36]. Although our study demonstrated a correlation between vBMD and bone strength, various factors affecting bone strength, such as microarchitecture and bone matrix composition, may differ according to ethnicity and gender, limiting the generalizability of our findings to the broader population. Further studies involving more diverse populations in terms of ethnicity and gender are necessary to generalize the findings to a global context. Second, there is the potential for measurement inaccuracy in defining the region of interest (ROI) for the box bone. However, since the box bone has a relatively rectangular shape, ROI delineation was straightforward and we considered the measurement error to be negligible. Third, the reproducibility of DECT-derived vBMD may be limited due to differences in CT scanner manufacturers and protocols. Calibration and measurement methods may vary between devices, potentially affecting vBMD values. Nonetheless, minor adjustments tailored to each scanner could standardize candidate selection, making clinical application feasible. Fourth, the Estimated Withstanding Strength (EWS) was calculated using the cross-sectional area of the distal femoral cutting surface, whereas the indentation test was performed on the box bone. This mismatch occurred because it was challenging to obtain bone specimens from the distal femur with sufficient thickness and consistent quality. After distal femoral cutting, only rounded dome-shaped bone with a maximum height of 9 mm can be obtained. This makes it technically challenging to prepare specimens with consistent 6 mm thickness. Therefore, we utilized box bone, which allowed for obtaining specimens of consistent quality. Accordingly, we measured the vBMD of the box area for analysis. Box bone generally exhibits weaker bone strength compared to the distal femoral cutting surface, which is closer to the weight-bearing area. Thus, when determining suitability of using box bone vBMD as a cut-off value, this mismatch would not increase the incidence of early failure. In addition, this study did not evaluate the tibial side, where subsidence more frequently occurs in cementless TKA. Despite this, the purpose of this study was to evaluate the correlation between DECT-derived vBMD and the actual bone strength, which was adequately addressed. This preliminary investigation demonstrates that a specific CT value can correspond to a particular level of bone strength, although further research with more refined parameters that incorporates tibial bone strength is needed. Furthermore, factors such as trabecular structure and bone matrix composition that could influence our experimental results were not evaluated in this study. We acknowledge that future research incorporating these factors would be valuable and could provide a more comprehensive assessment of bone strength. Nevertheless, we believe our findings remain meaningful as a preliminary effort to validate the diagnostic utility of DECT-derived vBMD. Finally, we defined the Minimum Required Strength (MRS) as 2.5 times body weight based on prior studies suggesting this threshold is sufficient for walking and daily activities [25,26]. In establishing the MRS, only patient weight was considered, without accounting for surgical factors such as malalignment or joint instability, which could increase the load beyond expected levels. Additionally, young, active patients tend to engage more frequently in activities such as stair climbing and squatting after TKA. Previous studies have reported that stair ascending generates approximately three times body weight, stair descending approximately 3.5 times body weight, and squatting approximately 2.5 to 3 times body weight loading. When determining the suitability for cementless TKA in young active patients, establishing different cut-off values considering these factors may be a more appropriate approach. However, this study was a preliminary investigation to confirm the diagnostic value of DECT, and differences according to patient populations or surgical factors were not incorporated into the study. The MRS of 2.5 times body weight proposed in this study is a conservative estimate established to confirm the diagnostic value of DECT vBMD. Further research that incorporates these additional factors will be required to more accurately identify suitable candidates for cementless TKA. Despite these limitations, this is the first study to demonstrate the correlation between DECT-derived vBMD and the actual bone strength around the knee and its practical value for identifying suitable candidates for cementless TKA.

## 5. Conclusions

This study demonstrates that DECT-derived vBMD is strongly correlated with the actual bone strength around the knee joint and thus serves as a reliable tool for assessing knee bone strength. In addition, it accurately predicts the adequate bone strength for cementless TKA, demonstrating excellent diagnostic performance.

## Figures and Tables

**Table 1:** Patient demographics and preoperative conditions *.

Parameters	Values (n = 190)
Demographic data	
Age (year)	68.1 ± 5.4 (53~86)
Gender (female)	155 (82)
Height (cm)	155.5 ± 7.0 (143.1~177.2)
Weight (kg)	65.7 ± 9.6 (45.4~94.3)
BMI (kg/m^2^)	27.2 ± 3.4 (19.6~36.5)
Diagnosis of osteoporosis †	
Normal (T score > −1.0)	67 (35)
Osteopenia (−1.0 ≤ T score ≤ −2.5)	95 (50)
Osteoporosis (T score < −2.5)	28 (15)
DXA (T-score)	
Mean lumbar spines	−0.5 ± 1.5 (−3.6 ~ 4.9)
Femur neck	−1.2 ± 1.1 (−3.5 ~ 2.8)
Volumetric BMD (mg/cm^3^)	
Axial image	55.9 ± 31.9 (−1.7~146.4)
Sagittal image	50.2 ± 26.9 (−1.2~115.3)
Coronal image	54.6 ± (−1.6~123.0)
Femoral component size †	
1	6 (3)
2	29 (15)
3	81 (43)
4	48 (25)
5	18 (10)
6	8 (4)

* Data are presented as mean ± SD (minimal ~ maximal value), except for gender, which is presented as the number of female patients and their percentages in parentheses; † data are presented as numbers of patients with their proportions in parentheses; BMI = body mass index; DXA = dual X-ray absorptiometry; BMD = bone mineral density.

**Table 2:** Biomechanical properties of the resected bone fragment *.

Parameters	Values
1st peak force (N)	59.5 ± 38.2 (6.7~202.5)
Compressive displacement at 1st peak force (mm)	0.9 ± 0.3 (0.3~1.8)
Maximal force (N)	81.2 ± 41.9 (14.6~244.0)
Compressive displacement at maximal force (mm)	1.8 ± 0.3 (0.4~2.0)
Stiffness (N/mm)	111.1 ± 80.2 (12.0~533.8)

* Data are presented as mean ± standard deviation (minimal value ~ maximal value).

## Data Availability

The data that support the findings of this study are available upon reasonable request from the corresponding author.
